# *Notes from the Field*: Rapid Linkage of a *Salmonella* Livingstone Outbreak to a Restaurant, Using Open-Ended Interviews and Patient Purchase Histories — Utah, 2023–2024

**DOI:** 10.15585/mmwr.mm7323a4

**Published:** 2024-06-13

**Authors:** Clarissa Keisling, Jennifer Hatfield, Delaney Moore, Savannah Graves, Brooke Smith, Jenni Wagner, Ravyn Casey, Erin L. Young, Kelly Oakeson, Willy Lanier

**Affiliations:** ^1^Council of State and Territorial Epidemiologists, Atlanta, Georgia; ^2^Utah Department of Health and Human Services; ^3^Utah County Health Department, Provo, Utah; ^4^Utah Department of Agriculture and Food; ^5^Career Epidemiology Field Officer Program, CDC.

**Summary**

**What is already known about this topic?**

Reported outbreaks of *Salmonella* Livingstone infection are rare. In one previously reported outbreak of *S. *Livingstone infection involving 60 patients, 54 reported gastroenteritis, seven of whom also reported a urinary tract infection (UTI); four others reported symptoms of UTI only.

**What is added by this report?**

During December 1, 2023–January 9, 2024, routine enteric disease surveillance identified *S.* Livingstone infections in five residents of two neighboring Utah counties. Open-ended interviews and review of purchase histories linked 11 cases to a local restaurant, which was closed in a timely manner. Four nonstool specimens (three urine and one blood) yielded the outbreak strain.

**What are the implications for public health practice?**

Incorporating open-ended interviews and purchase histories in foodborne illness outbreak investigations can expedite source identification and response.

During December 1, 2023–January 9, 2024, the Utah Department of Health and Human Services identified *Salmonella* Livingstone isolates from five residents living in two neighboring counties through routine enteric disease surveillance (*1*). Isolates were genetically similar by core-genome multilocus sequence testing (cgMLST). No related isolates from other states were reported to the National Center for Biotechnology Information, and none of the patients reported traveling outside the state during the week before illness, suggesting a local exposure. During initial, routine interviews, patients were asked about potential exposures, including restaurants, but did not report a common exposure. Health officials investigated to identify the source and prevent additional illnesses. This activity was reviewed by CDC, deemed not research, and was conducted consistent with applicable federal law and CDC policy.*

## Investigation and Outcomes

Beginning January 16, 2024, health officials conducted follow-up, iterative, open-ended interviews with patients and collected restaurant purchase histories to identify exposures during the week before illness onset.^†^ By January 17, four patients had reported eating at restaurant A; by January 19, a total of eight confirmed or probable cases had been identified through routine enteric disease surveillance, and all patients had reported eating at the same restaurant (Figure). No common meal was reportedly consumed at restaurant A. To prevent additional cases, public health officials closed restaurant A on January 19. On January 22, local and state officials collected 71 environmental and food samples from restaurant A and interviewed and collected stool samples from all nine employees for polymerase chain reaction testing, culture, and genomic sequencing.

**FIGURE:**
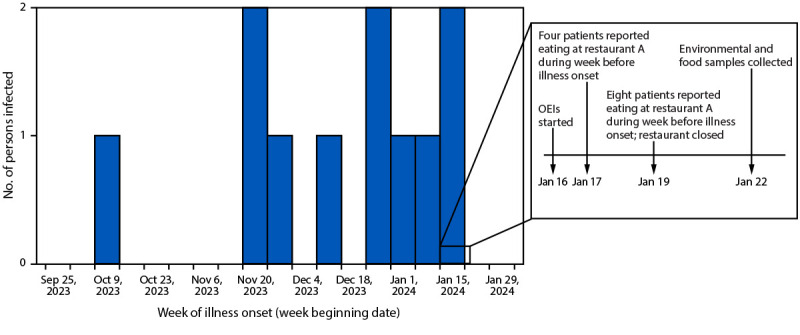
Number of persons infected with *Salmonella* Livingstone, by date of illness onset, response timeline, and information obtained through open-ended interviews — Utah, October 13, 2023–January 22, 2024* **Abbreviation**: OEI = open-ended interview. * By January 17, four patients had reported eating at restaurant A during the week before illness onset. By January 19, a total of eight patients (four additional patients) had reported eating at restaurant A during the week before illness onset. By February 9, all 11 patients had reported eating at restaurant A during the week before illness onset.

The outbreak strain was isolated from seven composite environmental swab samples (cleaning equipment; three-compartment sink and washing machine; drying rack, wooden stools, and trash can; utensils shelf; stove handles; sauce bottles; and outdoor dumpster) as well as from two composite food samples (sauces from grill station and vegetables and other ingredients from ingredient preparation area).

A case was defined as an infection with the outbreak strain of *S*. Livingstone, with illness onset on or after October 1, 2023. Overall, 11 cases were identified with illness onset during October 13, 2023–January 20, 2024; all patients reported eating at restaurant A during the week preceding illness onset (Figure).^§^ Reported gastrointestinal symptoms included diarrhea (10), abdominal pain (seven), vomiting (five), nausea (four), and bloody diarrhea (two). Median patient age was 45 years (range = 25–68 years); 55% of cases occurred among males. Six patients sought treatment at an emergency department, two of whom were hospitalized; no deaths were reported. Seven of the 11 patients received antibiotic therapy. The outbreak strain was isolated from nonstool specimens (three urine and one blood) from four patients.

The three patients with a urinary tract infection (UTI) included one man and two women. The two female patients with a UTI did not submit stool specimens, and one did not report gastrointestinal symptoms. The positive blood specimen was collected from a patient who reported diarrhea, fever, and neck stiffness; the outbreak strain was also isolated from this patient’s stool. Among five patients who reported the specific date and meal they ate at restaurant A (i.e., lunch or dinner) as well as the date their illness began, the median incubation period was 52 hours (range = 7–76 hours). The outbreak strain was isolated from the stool of one employee who began working at restaurant A on January 16; this employee reported eating multiple meals there and developed symptoms on January 20.

## Preliminary Conclusions and Actions

Although initial, routine patient interviews did not identify a common exposure, open-ended interviews and patient purchase histories enabled prompt identification of a restaurant source and led to closure of the restaurant within 3 days. Most patients reported symptoms of gastroenteritis. Three patients (27%) developed a UTI, one of whom reported only symptoms of UTI, an observation consistent with a previously reported outbreak (*2*). UTIs caused by non-typhoidal *Salmonella* are rare (*3*). In addition, one patient developed bloodstream infection. Environmental and food sampling results confirmed restaurant A as the outbreak source, suggested widespread contamination in the restaurant, and guided cleaning and sanitation; however, employee interviews did not identify a method by which the pathogen might have been introduced to the restaurant. Restaurant A re-opened on January 29; as of June 10, 2024, no additional infections with the outbreak strain have been reported. Using open-ended interviews and purchase histories in foodborne illness outbreak investigations can hasten source identification and response.

## Footnotes

* 45 C.F.R. part 46.102(l)(2), 21 C.F.R. part 56; 42 U.S.C. Sect. 241(d); 5 U.S.C. Sect. 552a; 44 U.S.C. Sect. 3501 et seq.

† Compared with initial interviews, follow-up interviews were loosely structured, with more open-ended questions regarding the patients’ activities and food consumption during the week preceding illness onset. Open-ended questions initially focused on nontraditional sources such as pet food, animal contact, or nonfood consumables.

§ The three additional patients were identified through routine enteric disease surveillance; these patients reported eating at restaurant A during the week before illness onset during initial interviews.
